# Preparation of a Major Metabolite of Iguratimod and Simultaneous Assay of Iguratimod and Its Metabolite by HPLC in Rat Plasma

**DOI:** 10.22037/ijpr.2019.1100641

**Published:** 2019

**Authors:** Jun-ping Han, Zhen-han Zhu, Yue-zhang Wu, Wen Qian, Zhi-yu Li, Miyu Nishikawa, Toshiyuki Sakaki, Chang-qing Yang

**Affiliations:** a *Department of Clinical Pharmacy, School of Basic Medicine and Clinical Pharmacy, China Pharmaceutical University, No. 639 Longmian Avenue, Jiangning District, Jiangsu Province, PR China.*; b *Department of Pharmacy, The Second Affiliated Hospital of Soochow University, Suzhou City, Jiangsu Province, 215004, PR China. *; c *Nanjing BRT-Biomed Co., Ltd. Jiangning District, Jiangsu Province, PR China. *; d *Department of Pharmaceutical Chemistry, School of Pharmacy, China Pharmaceutical University, No. 639 Longmian Avenue, Jiangning District, Jiangsu Province, PR China.*; e *Department of Biotechnology, Faculty of Engineering, Toyama Prefectural University, 5180 Kurokawa, Imizu, Toyama 939-0398, Japan.*

**Keywords:** Iguratimod, Recombinant human CYP450s, Metabolic pathway, Pharmacokinetics, HPLC assay

## Abstract

Iguratimod is a new synthetic disease-modifying antirheumatic drug intended to treat patients with rheumatoid arthritis. A new method using recombinant human CYP450s yeast cells containing c-DNA expressed P450s was applied to identify the metabolic pathways of iguratimod and to prepare its metabolite. The metabolite was isolated, and its structure was identified by quadrupole time-of-flight-mass spectrometry and nuclear magnetic resonance. Furthermore, a selective and sensitive high performance liquid chromatography (HPLC) method was developed for the simultaneous quantification of iguratimod and its major metabolite in rat plasma for the first time. The results indicated that iguratimod was mainly metabolized to a metabolite by CYP2C9 and CYP2C19 in *in-vitro* study. The structure of the metabolite was identified as M2 (N-[3-(acetamido)-4-oxo-6-phenoxy-4H-chromen-7-yl]methanesulfonamide). HPLC assay was achieved on a C18 column using methanol-water containing 0.1% trifluoroacetic acid (55:45 v/v) at a flow rate of 1 mL/min with UV detection at 257 nm. Standard calibration curves were obtained in the concentration range of 0.5–20 µg/mL for iguratimod and its metabolite M2. The lower limits of detection of iguratimod and M2 in rat plasma were 0.1 and 0.25 µg/mL, respectively. The intra- and inter-day precision (RSD%) were within 5% for the two analytes. The average recoveries of the analytes were greater than 90%. In conclusion, recombinant human CYP450s whole-yeast transformation system could be successfully used to identify and prepare the major metabolite of iguratimod. The HPLC method we developed could be successfully applied to evaluate pharmacokinetics of iguratimod and its metabolite M2 in rats.

## Introduction

Iguratimod, a novel disease-modifying antirheumatic drug, which is now used in hospitals in China and Japan, has been confirmed as a highly efficacious and safe drug for rheumatoid arthritis therapy (1). Previous studies on iguratimod mainly focused on its clinical efficacy, mechanisms and adverse reactions since it was firstly used to treat rheumatoid arthritis. However, there were few researches on the pharmacokinetics of iguratimod.

Iguratimod is extensively metabolized by the liver, and several metabolites of iguratimod have been characterized in plasma from healthy male subjects following oral administration of the drug (Figrue 1) (2). Previous researches reported that the major metabolites of iguratimod are a deformylated form (M1) and its N-acetylated form (M2). The other metabolites are phydroxylated forms in the 6-phenoxy groups of iguratimod, M1, and M2, which have been termed M4, M5, and M3, respectively (2). However, none of the studies confirmed the specific metabolic pathways of iguratimod. A high-performance liquid chromatographic (HPLC) method with UV detector had been developed for the determination of iguratimod (T-614) in rat plasma (3). The *in-vivo* pharmacokinetics of iguratimod was consistent with the dynamics of the one-compartment model. The results of a preclinical pharmacokinetic study indicated that T-614 was absorbed rapidly and eliminated slowly in animals (4). However, none of studies had developed a method of simultaneous determination of iguratimod and its metabolite in rat plasma.

Elucidation of phase I metabolic pathways and preparation of the metabolites of new drugs are very important in the drug discovery and the clinical use of new drugs. Enzyme inhibition or induction is known to alter the activity of CYP isozymes and potentially increase the risk of side effects or toxicity of drugs. Metabolite standards of the new drug are of particular importance in the context of metabolites in safety testing (MIST). Meanwhile, metabolite standards are needed as analytical standards for the respective bioanalytical assay procedures, but they are often not commercially available, and their classical chemical synthesis can be cumbersome (5). *In-vitro* models of drug metabolism include subcellular cell models (such as microsomes), cell line models (such as primary hepatocytes, transgenic cell lines, stem cell-induced differentiation of liver cells), recombinant metabolic enzyme models, tissue sections, *in-vitro* animal model (such as liver/intestinal perfusion model) and so on (6). Drăgan CA *et al.* successfully prepared 4’-hydroxydiclofenac at gram-scale from diclofenac using a fission yeast strain functionally co-expressing human CPR and CYP2C9. They demonstrated that recombinant human CYP450s whole-cell yeast transformation system could be used in the confirmation of metabolic pathways and preparation of drug metabolite standard (7).

In this study, we investigated the metabolic pathways of iguratimod and prepared a main metabolite of iguratimod using a recombinant human CYP450s yeast whole-cell transformation system. The research method will provide a reference for studies on metabolic pathways and preparation of metabolites of new drugs or candidate drugs. In addition, an HPLC method for the simultaneous determination of iguratimod and its metabolite M2 in rat plasma was developed for the first time. This method will provide a basis for clinical pharmacokinetic studies of iguratimod and its metabolite M2.

## Experimental

*Materials*

Iguratimod (T-614, purity 99.2%, HPLC grade) was supplied by Jiade Medical Technology Co., Ltd. (Changzhou, China). Iguratimod metabolite (M2) was isolated and purified in our laboratory (purity >98%, HPLC grade). Iguratimod tablets were purchased from Simcere (Hai’kou, Hainan, China). Phenacetin (purity ≥98%), used as the internal standard (IS), was purchased from Macklin Biochemical Co., Ltd. (Shanghai, China). Recombinant human CYP450s yeast cells containing c-DNA expressed P450s (CYP1A2, CYP2C9, CYP2C19, CYP2D6, CYP2E1, and CYP3A4) were obtained from Nanjing BRT-Biomed Co., Ltd. (Nanjing, China). Other chemicals were obtained from the following sources: D-(+)-Glucose from Aladdin Industrial Corporation (Shanghai, China); di-Potassium hydrogen phosphate trihydrate and Potassium dihydrogen phosphate from Xilong Chemical Co. Ltd. (Shantou, Guangdong, China); trifluoroacetic acid (TFA) from Nanjing Chemical Reagent Co., Ltd. (Nanjing, China); Demethyl sulfoxide from Sinopharm Chemical Reagent (Nanjing, China); Ethyl acetate from Nanjing Chemical Reagent Co., Ltd. (Shanghai, China). Acetonitrile (HPLC grade) and methanol (HPLC grade) were purchased from Tedia (Fairfield, OH, USA). Distilled water, prepared from demineralized water, was used throughout the study.

*Chromatographic condition*

The chromatographic system was performed by LC-2010A HT high performance liquid chromatograph (Shimadzu, Japan). The assay was performed on ACE-C18 column (250 × 4.6 mm × 5 μm) (ACE, UK) and a InertSustain C18 precolumn (4.0 × 10 mm × 5 μm) (Shimadzu, Japan) was fitted just before the inlet junction of the analytical column. The mobile phase consisted of methanol-water containing 0.1% TFA (55:45 v/v) at a flow rate of 1 mL/min with UV detection at 257 nm. The column temperature was maintained at 30 °C, and the injection volume was 10 μL. 

*Incubation of iguratimod with the recombinant human CYP450s yeast cells*

The incubation mixture (4 mL final volume) of recombinant CYP450s yeast whole-cell transformation system includes 1 g recombinant CYP450s yeasts containing c-DNA expressed P450s (CYP1A2, CYP2C8, CYP2C9, CYP2C19, CYP2D6, CYP2E1 or CYP3A4), 2 mL 0.2 M K_2_HPO_4 _phosphate buffer (pH 7.4), 0.4 mL 20% D-(+)-Glucose solution, 1.4 mL sterile water and 0.2 mL 10 mM iguratimod dissolving in DMSO to make the final system consisting of 2% glucose solution and 0.5 mM iguratimod. Seven types of recombinant human CYP450s yeast whole-cell reaction system at seven individual 50 mL tubes were initiated at the parallel time. The mixture in each tube was incubated for 72 h at 30 °C. The samples were collected from the incubation system at 0, 24, 48, and 72 h by taking 100 uL reaction solution to 1.5 mL clean and dry tubes and adding 200 μL methanol. The samples were fully extracted by vortex-mixing for 2 min. After centrifugation at 12,000×g for 10 min, the supernatant was collected and an aliquot (10 μL) was injected into the chromatographic system for analysis.

*Identification of the iguratimod metabolite in rat plasma sample*

Male Sprague-Dawley rats (180-200 g) were obtained from Jiesijie experimental animal Co., Ltd. (Shanghai, China) (license key, SCXK 2013-0006) and housed with a 12 h light/12 h night cycle at ambient temperature (about 25 °C) and 60% relative humidity. Free access to food and water was allowed at all times except for fasting 12 h before the experiment but with free access to water. All animal experiments were carried out according to the Guidelines for the Care and Use of Laboratory Animals, and were approved by the Animal Ethics Committee.

To a 50 μL aliquot of rat plasma, 25 μL Phenacetin IS solution (20 μg/mL) and 25 μL acetonitrile were added. After vortex-mixing for 2 min and centrifugation (12,000×g) for 10 min, the supernatant was collected and an aliquot (10 μL) was injected into the chromatographic system for analysis.

*Preparation and isolation of the iguratimod metabolite*

To prepare the metabolite of iguratimod, recombinant human CYP2C9 yeast cell reaction system was used according to the method described above. Total volume of the system is 80 mL which contains 20 g recombinant CYP450s yeasts, 40 mL 0.2 M K_2_HPO_4 _phosphate buffer (pH 7.4), 8 mL 20% D-(+)-Glucose solution, 28 mL sterile water and 4 mL 10 mM iguratimod dissolving in DMSO. After incubation at 30 °C for 72 h, 160 mL acetic ether were added to stop the reaction. Then the acetic ether extraction solution was concentrated by a rotary evaporator and then redissolved using 10 mL methanol. Preparative HPLC was performed in an isocratic elution mode using Durashell C18 (250 mm × 10 mm, 10 μm). The isocratic mobile phase of water-methanol (52:48, v/v) containing 0.1% TFA (v/v) was run at a flow rate of 7 mL/min. Other parameters were as follows: detection wavelength, 257 nm; injection volume, 200 μL; column and ambient temperature, 25 °C. The eluents were freeze-dried by a Bilon freeze dryer to prepare the metabolite powder.

*Identification of the iguratimod metabolite structure by QTOF-MS and Nuclear magnetic resonance spectroscopy*

HR-MS spectral data was obtained on Agilent technologies 6520 Accurate-Mass QTOF-MS instruments (Agilent, USA). Data acquisition was performed with Agilent Mass hunter Workstation with advanced data acquisition and data analysis capabilities. The mass spectrometer was operated in the ESI-positive mode, and the MS data were collected at the range of 320 to 490. The HNMR spectra were recorded on Bruker AV-300 (300 MHz) apparatus (Bruker, USA).

**Figure 1:**
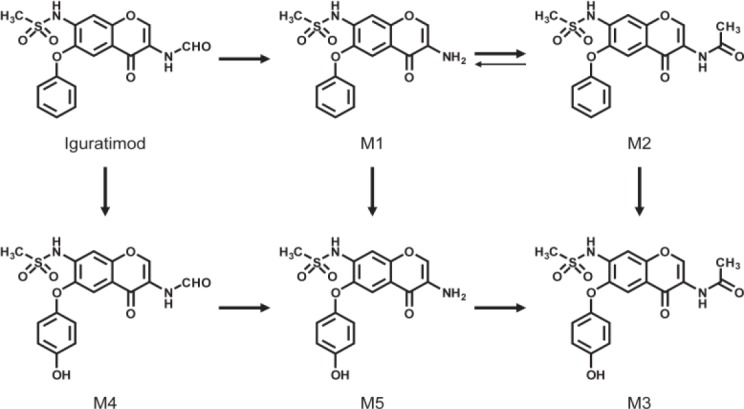
Metabolites and possible pathways of iguratimod

**Figure 2:**
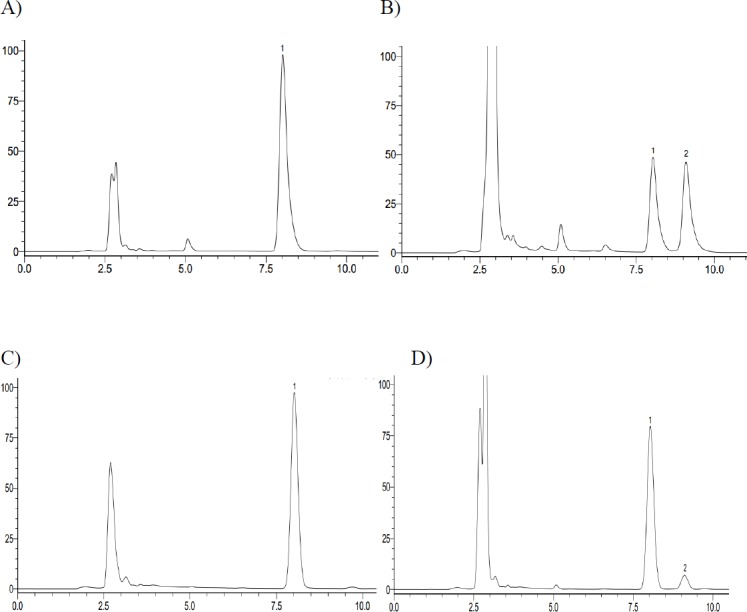
The representative HPLC chromatograph obtained from the analysis of reaction solutions in the recombinant human CYP2C9 and CYP2C19 reaction system after 72 h incubation. (A) CYP2C9 incubation – 0 h; (B) CYP2C9 incubation – 72 h; (C) CYP2C19 incubation – 0 h; (D) CYP2C19 incubation – 72 h (1. Iguratimod; 2. iguratimod metabolite)

**Figure 3:**
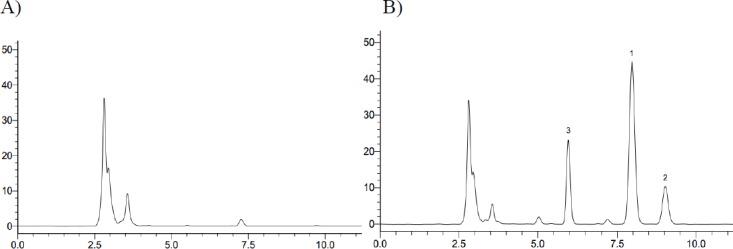
Typical HPLC chromatograph for iguratimod and its metabolite in rat plasma. (A) Rat blank plasma; (B) Rat plasma sample at 6 h after oral administration of iguratimod + Phenacetin (1. Iguratimod; 2. iguratimod metabolite; 3. IS, Phenacetin)

**Figure 4:**
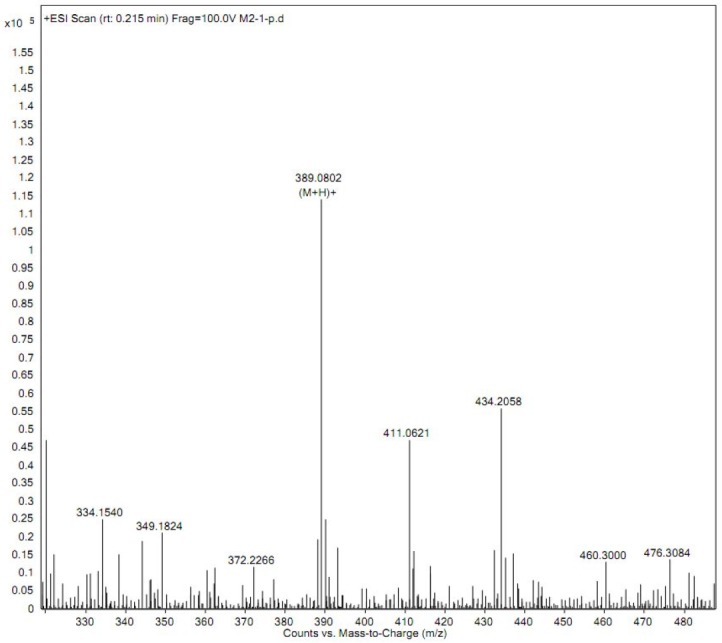
Product ion mass spectra of iguratimod metabolite

**Figure 5:**
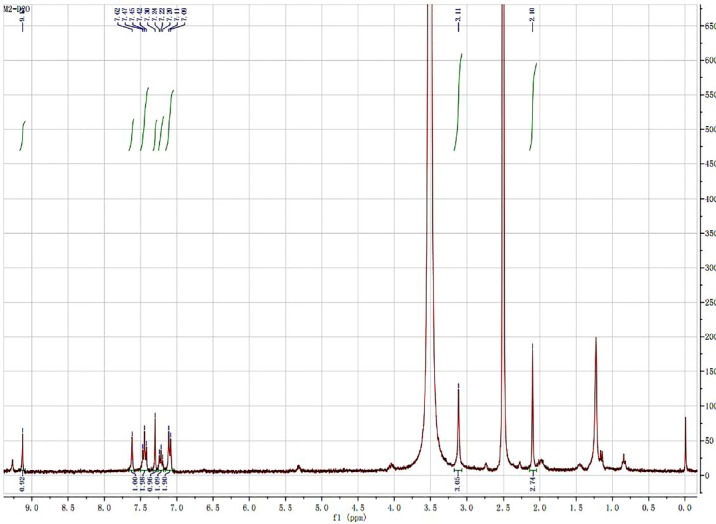
HNMR spectra of iguratimod metabolite

**Figure 6:**
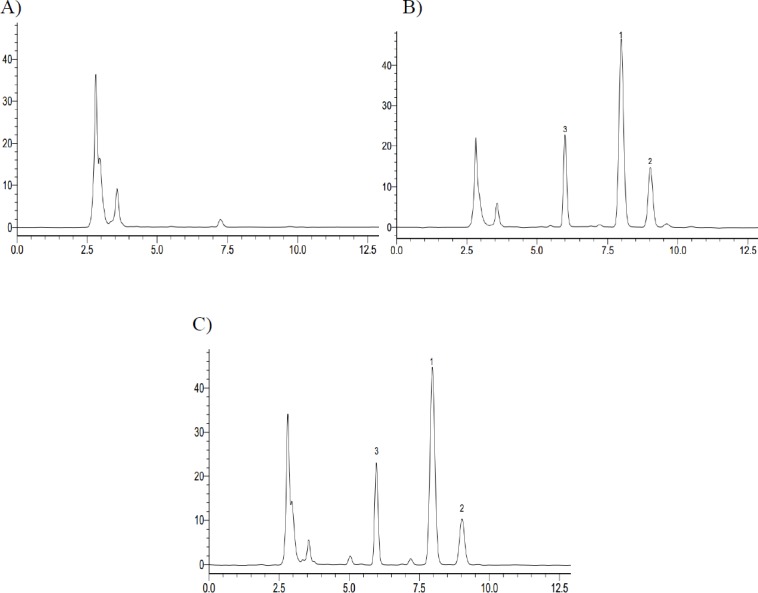
High Performance Liquid Chromatography of iguratimod, a iguratimod metabolite M2 and Phenacetin in Rat Plasma. (A) Blank plasma; (B) Standard plasma samples; (C) Plasma samples at 6 h after oral administration of iguratimod (1. Iguratimod; 2. iguratimod metabolite; 3. IS, Phenacetin)

**Figure 7:**
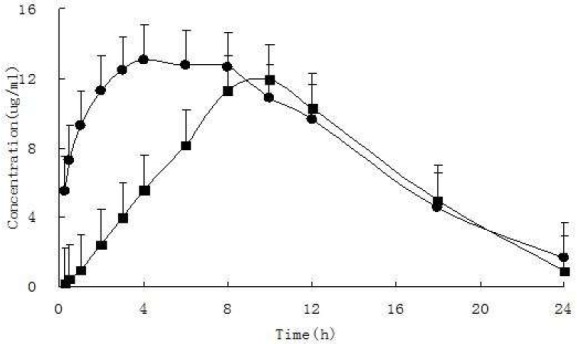
Mean plasma concentration-time profiles of iguratimod and iguratimod metabolite in rat plasma after oral administration of10 mg/kg iguratimod. (mean ± SD. n = 7). (●iguratimod; ■iguratimod metabolite M2)

**Table 1:** Precision, accuracy and extraction recoveries of analytes (n = 3).

**Analytes**	**Spiked (ug/mL)**	**intra-day**		**inter-day**		**Extraction Recovery**	
		**Precision (RSD%)**	**Accuracy (RE%)**	**Precision (RSD%)**	**Accuracy (RE%)**	**Mean (%)**	**Precision (RSD%)**
iguratimod	0.5	2.29	-1.75	2.36	-6.79	98.25 ± 0.01	2.29
	10	0.48	4.12	0.21	3.67	104.12 ± 0.50	0.48
	20	0.15	-2.75	1.16	-1.58	97.25 ± 0.03	0.15
iguratimod metabolite	0.5	1.56	-4.21	1.90	-4.95	95.79 ± 0.01	1.56
	10	0.52	-2.79	0.29	-2.69	97.21 ± 0.50	0.52
	20	0.56	-3.33	1.19	-2.55	96.67 ± 0.11	0.56

**Table 2:** Pharmacokinetic parameters of iguratimod and its major metabolite M2 in rat plasma (mean ± SD, n = 7)

**Parameters**	**units**	**iguratimod**	**iguratimod metabolite**
t1/2	h	5.12 ± 2.45	3.70 ± 0.85
Cmax	mg·L-1	14.16 ± 2.24	12.56 ± 3.58
Tmax	h	5.86 ± 2.19	9.14 ± 1.07
AUC0→24 h	mg·h·L-1	197.42 ± 16.58	151.79 ± 41.47
ClZ	L·h-1·kg-1	0.05 ± 0.00	0.07 ± 0.02
VZ	L·kg-1	0.34 ± 0.13	0.38 ± 0.19

*Method validation of HPLC*

To assess selectivity of the method, blank plasma samples obtained from seven rats were processed by the same extraction procedure and chromatographed to determine whether endogenous components would interfere with the determination of iguratimod, iguratimod metabolite, and internal standard (Phenacetin).

The Calibration curves were prepared by assaying standard plasma samples at six concentration levels of iguratimod and metabolite over the range of 0.5-20 ug/mL, respectively. The calibration curves were constructed by plotting the peak area ratio of analytes to IS, and concentrations being used as y and x variables in a standard regression analysis. The lower limit of detection was defined as the detected concentration when the S/N ratio is 3.

Precision and accuracy were carried out in three replicates at 0.5, 10, and 20 µg/mL on the same day and on three consecutive validation days. The accuracy was expressed as relative error (RE%). The precision was calculated by relative standard deviation (RSD%). Intra- and inter-day precisions calculated as RSD (%) were required to be below 15% and accuracy as RE (%) to be within ±15%. Recoveries of the iguratimod and iguratimod metabolite were determined by comparing post-extraction standard plasma concentrations and control standard samples at corresponding concentrations.

*Application to pharmacokinetic studies*

The studies were approved by the Animal Ethics Committee of China Pharmaceutical University. The animals were adapted to the facilities for one week, and then fasted with free access to water overnight prior to the experiment. The iguratimod tablets were suspended in 0.1% CMC-Na aqueous solution and administered to the rats (10mg/kg bodyweight) by oral gavage. Approximately, 300 μL blood samples were collected from fundus venous plexus before dosing and at 0.25, 0.5, 1, 2, 3, 4, 6, 8, 10, 12 and 24 h following oral gavage. The blood samples were transferred to heparinized Eppendorf tubes and centrifuged at 4000×g for 10 min to separate the plasma. The plasma samples were immediately frozen at -80 °C until analysis. The pharmacokinetic parameters of iguratimod and its metabolite were calculated using Phoenix WinNonlin^® ^6.1 version. All results were represented as mean ± SD.

## Results and Discussion

*Identification of the metabolic pathway of iguratimod*

In *in-vitro* study, iguratimod was incubated with seven types of recombinant human CYP450s (CYP1A2, CYP2C8, CYP2C9, CYP2C19, CYP2D6, CYP2E1, and CYP3A4) yeasts, respectively. After incubation at 30 °C for 72 h, the reaction solution was extracted with methanol and analyzed by HPLC. We found that there was a new chromatographic peak in the recombinant human CYP2C9 and CYP2C19 transformation system with the retention time at approximately 9.064 min (Figure 2). Meanwhile, the reaction rate of iguratimod in recombinant human CYP2C9 transformation system was higher than that in recombinant human CYP2C19 transformation system, with the value 50.73% and 15.83%, respectively. There were no new chromatographic peaks and the reaction rate was near 0% for the other five CYP450s transformation system. The reaction rate was calculated using the following equation:

Reaction rate%=iguratiimod concentration at 0h-iguratimod concentration at 72h iguratimod concentration at 0h×100%

In *in-vivo* study, we found that there was a same chromatographic peak in rat plasma after taking iguratimod, which was at the same retention time of approximately 9.064 min with that *in-vitro* study (Figure 3). 

There was a new chromatographic peak after 72 h incubation when assaying the recombinant human CYP2C9 and CYP2C19 reaction solutions using HPLC. In contrast, it was not observed in the recombinant human CYP1A2, CYP2C8, CYP2D6, CYP2E1, and CYP3A4 incubation system under the present conditions, as determined by HPLC with UV detection at 257 nm. Meanwhile, we found a same chromatographic peak at the same retention time in the rat plasma with that *in-vitro* study. Therefore, we expediently assumed that iguratimod could be metabolized by CYP2C9 and CYP2C19.

In the present study, we examined the metabolic pathways of iguratimod in *in-vitro* study using the recombinant human CYP450s (CYP1A2, CYP2C8, CYP2C9, CYP2C19, CYP2D6, CYP2E1, and CYP3A4) yeast whole-cell transformation system. It is the first time to discover the metabolic pathways of iguratimod using the transformation system in this study. Among *in-vitro* recombinant human metabolic enzyme expression system, yeasts have a more complete cell subcellular (endoplasmic reticulum) structure comparing to prokaryotic expression system. Meanwhile, yeast expression system has a relatively high expression rate and catalytic activity of recombinant enzyme, short production cycle, and it is suitable for large quantities of the preparation comparing to mammalian cell expression system (8). Yasuda *et al.* transfected human CYP1A1, 1A2, 2A6, 2B6, 2C8, 2C9, 2C18, 2C19, 2D6, 2E1 and 3A4 genes into yeast cells to prepare recombinant human CYP450s and investigated the metabolic characteristic of sesamin. They inferred that sesamin was metabolized by CYP2C9, 1A2, 2C19, and 2D6, mainly by CYP2C9 (9). Therefore, *in-vitro* recombinant human CYP450s yeast system is a novel and effective way for studying the metabolic characteristics of new drugs. 

*Isolation and identification of the iguratimod metabolite by QTOF-MS and HNMR*

Prior to structure identification, the metabolite was prepared from recombinant human CYP2C9 yeast transformation system using preparative HPLC method. The final reaction rate of iguratimod in preparation system was 43.5%. The yield of iguratimod metabolite was 15.4% after extraction and purification. The final purity of the iguratimod metabolite we prepared reached at 98.8%. The chemical structure was identified by the combination of QTOF-MS and HNMR. Figure 4 shows the QTOF-MS spectra of iguratimod metabolite, which formed protonated molecules [M+H] as major ion peaks. HRMS (ESI): m/z, calculated for C_18_H_16_N_2_O_6_S, found 388.0729 (M þ H) þ. Figure 5 shows the HNMR spectra of iguratimod metabolite. ^1^H NMR (300 MHz, DMSO-*d*_6_ + D_2_O): *δ* 9.13 (s, 1H), 7.62 (s, 1H), 7.45 (t, *J* = 7.6 Hz, 2H), 7.30 (s, 1H), 7.25 – 7.18 (m, 1H), 7.10 (d, *J* = 7.8 Hz, 2H), 3.11 (s, 3H), 2.10 (s, 3H). On this basis, the assignment of the iguratimod metabolite is M2 (N-[3-(acetamido)-4-oxo-6-phenoxy-4H-chromen-7-yl]methanesulfonamide) as shown in Figure 1.

The current reaction system that we prepared a metabolite standard of iguratimod, included recombinant CYP450s yeasts containing c-DNA expressed CYP2C9, 0.2 M K_2_HPO_4 _phosphate buffer (pH 7.4), D-(+)-Glucose solution, sterile water and iguratimod dissolving in DMSO. A previous literature had thoroughly investigated the influence of the factors such as pH, cell density and incubation time on the incubation for the whole-cell biotransformation of 4’-methyl-α-pyrrolidiobutyrophenone (MPBP) and dexamethasone (DXM) in CAD58 cultures (10). In our study, we found pH had no apparent influence on iguratimod transformation rate. The temperature of yeast cell culture is usually set at 30 °C; however, the optimum temperature for human liver drug enzyme is 37 °C. Therefore, we compared the reaction rate of iguratimod between 30 °C and 37 °C in the pre-study. It was found that the reaction rate of iguratimod was higher at 30° C than at 37 °C. Metabolite formation was linear in the initial phase and leveled off in later incubation phases, reaching almost zero after approximately 72 h. 

The structure of the metabolite prepared from recombinant CYP2C9 yeast incubation system was identified by QTOF-MS and HNMR. HRMS data show that m/z of iguratimod metabolite is 388.0729, calculated for C_18_H_16_N_2_O_6_S. On the basis of HRMS and HNMR data, we confirmed the structure of iguratimod metabolite as M2 (N-[3-(acetamido)-4-oxo-6-phenoxy-4H-chromen-7-yl]methanesulfonamide) (Figure 1). We inferred that iguratimod may be metabolized by CYP2C9 and CYP2C19 to M2. 

*Method validation of HPLC*

Typical chromatograms obtained from a blank, a spiked plasma sample with the analytes (at 10 µg/mL) and IS, and a plasma sample after an oral administration of iguratimod are shown in Figure 6. No obvious interferences from endogenous plasma substances were observed under the chromatographic conditions.

To evaluate linearity, calibration of iguratimod and iguratimod metabolite M2 (0.5–20 µg/mL) were prepared and assayed on three consecutive days. The peak area ratios of iguratimod or iguratimod metabolite to IS in rat plasma varied linearly over the concentration ranges. The regression equations for the drug and it’s metabolite were y = 0.2311x + 0.0321 (r = 0.9999) and y = 0.1337x – 0.0022 (r = 0.9999), respectively, where y refers to the peak area ratios of analytes to IS and x is the concentration of iguratimod or iguratimod metabolite. The present HPLC method gave the lower limit of detection for iguratimod and its metabolite at 0.1 µg/mL and 0.25 µg/mL, respectively.

Both intra- and inter-day precisions were <5.0% at three QC levels (0.5, 10.0 and 20.0 µg/mL), and RE values for iguratimod and iguratimod metabolite were within ±10%. The mean extraction recoveries of iguratimod at concentrations of the QC levels were 98.25, 104.12, and 97.25%, respectively. The mean extraction recoveries of iguratimod metabolite M2 at concentrations of the QC levels were 95.79, 97.21, and 96.67%, respectively. These results indicated that the present method has an acceptable precision and accuracy (Table 1).

Previous studies that conducted on this drug is just to determine the HPLC method of iguratimod in animal plasma and in the healthy adults plasma (4, 11). However, the determination of iguratimod metabolite data was not available in the previous study because iguratimod metabolite had not been studied. Therefore, a new HPLC method of simultaneous determination of iguratimod and its metabolite in rat plasma was developed for the first time in this study. No obvious interferences from endogenous plasma substances were observed under the chromatographic conditions (Figure 3). There was excellent correlation between the ratio of peak area and concentration for iguratimod and its metabolite within the test ranges. The assay precision, accuracy and extraction recoveries for iguratimod and its metabolite were within the acceptance (Table 1). This method was successfully applied to the pharmacokinetic studies of iguratimod and its metabolite after oral administration of iguratimod in rats (Figure 7 and Table 2).

*Application to pharmacokinetic studies*

The present HPLC method was successfully applied to determining the plasma concentration of iguratimod and iguratimod metabolite in Sprague–Dawley rats. After a single oral gavage administration of 10 mg/kg iguratimod to rats, the concentration–time profile was constructed for up to 24 h. Figure 7 shows the mean concentration–time profile of iguratimod and iguratimod metabolite in the rat plasma. The pharmacokinetic parameters for the drug and its metabolite M2 are shown in Table 2.

## Conclusion

Our study demonstrated that M2 is a major metabolite of iguratimod in phase Ⅰ metabolism, and iguratimod is mainly metabolized by CYP2C9 and CYP2C19, especially by CYP2C9. Meanwhile, we developed a highly selective and reproducible HPLC method for simultaneous determination of iguratimod and its metabolite in rat plasma. The method could be useful for pharmacokinetic study in rats. In this experiment, the metabolic pathways of iguratimod were verified just in 7 recombinant human CYP450s (CYP1A2, CYP2C8, CYP2C9, CYP2C19, CYP2D6, CYP2E1, and CYP3A4) whole yeast cell reaction systems. These metabolic enzymes are the metabolic subtypes of most drugs. However, there may be other phase I metabolic pathways for iguratimod, which needs further investigations.
